# Students tell us what good written feedback looks like

**DOI:** 10.1002/2211-5463.12841

**Published:** 2020-03-30

**Authors:** Susanne Voelkel, Tunde Varga‐Atkins, Luciane V. Mello

**Affiliations:** ^1^ School of Life Sciences University of Liverpool UK; ^2^ Centre for Innovation in Education University of Liverpool UK; ^3^ School of Life Sciences University of Liverpool UK

**Keywords:** effective feedback, feedback, feedforward, student perceptions

## Abstract

Feedback can be an important element of learning, but only if students engage with it. Students are only likely to engage with feedback that they find useful. This study aimed to identify characteristics of written feedback perceived by students as effective. We used a mixed‐method approach, integrating quantitative and qualitative data that were collected through the analysis of feedback that was identified by students as good, a student questionnaire, as well as interviews and a focus group exploring students’ views on what good feedback looks like. Although the results show that length and composition of ‘good’ feedback can be extremely variable, some common characteristics could be identified, leading to a set of recommendations for staff marking written assessments. According to students, good feedback should be detailed and specific, and it should tell students how they can improve. Students also find it important that feedback is honest and constructive. In addition, positive reinforcement was identified as important by the focus group, although few examples of good written feedback on the assignment contained any direct praise. Surprisingly, feedforward which might help students in other modules did not feature highly in students’ perceptions of good feedback, possibly indicating a focus by students on improving the current assignment rather than on future assignments.

## Graphical abstract

We all want to do our best when it comes to providing feedback to students. But what does ‘good’ feedback look like? In this study, we explore the characteristics of written feedback that was perceived as good feedback by students. Based on these ‘good feedback’ characteristics and student views, we provide some recommendations on how to give good feedback.

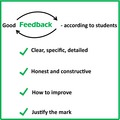

**Abbreviations**
HE: higher education
NSS: National Student Survey

This study intends to identify characteristics of written feedback that is seen as good feedback by students. Students in higher education (HE) expect to receive feedback on their work [1]. If feedback is not provided, or if it does not meet their expectations, students express their dissatisfaction in module evaluations and in central student surveys such as the UK National Student Survey (NSS). The NSS is an annual survey for final year undergraduate students aimed to gather feedback on their experiences during their course. Although overall scores for feedback have improved in recent years [2], feedback has consistently been scoring lower than most other course features in the UK NSS [3, 4]. As a consequence, many HE institutions strive to improve their ratings in this area [5].

In addition to the importance of NSS ratings for UK HE institutions, there are good reasons for improving feedback to students. It is generally agreed that feedback can have a significant beneficial effect on student learning [6, 7]. But this can only happen if feedback is of high quality, if students read and understand their feedback, and if they act on it [8, 9, 10]. Unfortunately, these conditions are not always met. It appears that students often do not collect their feedback [11, 12, 13]. Other studies have shown that even if they collect and read their feedback, students do not always understand their tutors’ comments [1, 14, 15]. Many students find feedback comments vague [16] and lacking advice for future assignments [17]. According to Price *et al*. [18], ‘a key reason for assessment failing to support learning is ineffective feedback’.

So, what is the advice for a tutor/assessor who aims to give good feedback? Nicol and Macfarlane‐Dick [19] proposed ‘seven principles of good feedback practice’ in the context of formative assessment, aiming to help the student to ‘take control of their own learning’. Nicol [4] suggested that written feedback comments should be (among other things) understandable, specific, timely, balanced and forward‐looking. Regarding the latter, Boud and Molloy [9, p. 702] even argue that ‘feed forward [is] not a separate notion but necessary characteristic of feedback’.

However, tutors appear to struggle putting this advice into practice. Carless [20] found discrepancies between tutors’ perceptions about the feedback they are giving, and what students thought about it. For example, tutors believe they are providing more detailed and useful feedback than student think they do. Other studies have shown that tutors have a variety of differing beliefs of the role of feedback [1, 17]. For example, some tutors see the main purpose of giving feedback as justifying the grade, others wish to advise students, and some think that good students do not need detailed feedback at all [1]. What is more, Orrell [21] found that there can be a difference between what staff believe feedback should look like, and what feedback they actually provide. Reasons for these problems could include the fact that although the majority of academics try to do their best to provide helpful feedback to students [22], many practitioners have little formal educational training [23, 24]. Also, Orrell [21] points out that there is more emphasis on the summative than on the formative role of assessment, leading to feedback that may be ‘defensive’ (i.e. a justification of the mark) rather than learning‐oriented.

Student expectations and perceptions of feedback are critical for their engagement with it [17, 25]. Tutors tend to think that students are only interested in marks, but most students want to improve and are interested in tutors’ views of their work [20, 26]. Students do not always completely agree on what useful feedback is [27]. For example, Orsmond and Merry [28] found differences between high‐ and low‐achieving students in terms of feedback perceptions. However, in general, feedback is seen as unhelpful if it lacks detail and does not contain suggestions for improvement [29]. The ability and willingness of students to engage with feedback depends on the extent to which they understood feedback and their self‐efficacy. In addition, the opportunity to resubmit a piece of work provides strong motivation for students [17].

Arguably, the most common feedback provided to students consists of written comments on student coursework assignments [1]. Recently, more and more written feedback is being provided electronically. Although paper‐based feedback comments have a lot of similarities with online feedback [30], sending feedback electronically is an effective and simple means of communicating feedback to students and can enhance the way in which they receive and engage with it [31]. Advantages include flexibility and convenience for the student regarding when and where they access the feedback, and the increased efficiency that allows a more timely feedback distribution [32]. Bridge and Appleyard [33] reported that most students preferred online submission of assignments and online feedback over hard copies. Others found that online submission and feedback can have negative sides, including a depersonalisation and unexpected technical difficulties [15, 34]. However, Hast and Healy [15] found an increasing trend in preference and it seems likely that electronic coursework submission and feedback are here to stay. An additional benefit of this approach is the opportunity to monitor and share feedback practice. As Hounsell [3] points out, we need to know more about content and quality of feedback comments within teaching teams in order to identify problems and share good feedback exemplars.

The aim of this study was to identify common characteristics of good feedback and based on that to create guidance for biosciences tutors/assessors on how to produce effective written feedback to students. In the context of this study, the notion of good (effective) feedback is entirely based on student perceptions. This is in line with the idea that students are only likely to engage with what they perceive as high‐quality feedback [11]. To identify common characteristics of good feedback, we used four different methods: (a) analysis of written feedback examples that were provided by students who had been asked to send us written electronic feedback that they found effective; (b) a student questionnaire; (c) interviews; and (d) a focus group. This triangulated study design resulted in rich data and allowed us to produce a set of recommendations for assessors.

## Methods

This study was funded by a Learning and Teaching Fellowship at the University of Liverpool, UK, awarded to one of the authors (SV) in 2016. Ethics approval was granted by the University’s Ethics Committee on 19 July 2016. The study followed a mixed‐method approach using qualitative and quantitative results from the analysis of examples of good feedback (i.e. feedback perceived as useful by students), a questionnaire, interviews and a focus group, thereby using a high degree of triangulation. All participants were undergraduate second‐ and third‐year students in the School of Life Sciences. Students were provided with information sheets prior to their participation, and participants signed consent forms for interviews and the focus group. As an incentive, all interview and focus group participants received a £10 voucher.

### Feedback analysis and questionnaire

This part of the study was conducted between September and December 2016 and comprised two elements: quantitative and qualitative analysis of written feedback examples and a short questionnaire. All second‐ and third (final)‐year students (cohort size: 420 and 400, respectively) were invited by email to participate in the study if they thought that they had received particularly good feedback for a specific written assignment (see below). Respondents were also asked to complete a questionnaire asking them (a) to explain what they liked about their feedback, (b) how they responded to it and (c) to suggest any further improvement of the received feedback.

The study focused on two written assignments that were completed by all second‐ and third‐year students, respectively, via an online marking tool (Turnitin). For second‐year students, the assignment consisted of a 1500 word essay. After receiving marks and feedback, students had the opportunity to resubmit a revised essay if they wanted to improve their essay mark. The third‐year students’ assignment was a 3000 – word literature review as part of their final year research project. Although the third‐year students could not resubmit their work to achieve a better mark, feedback for the literature review would then help students to write the introduction to their research project report that was due to 5 months later. For both assignments, a marking rubric was used in addition to the written feedback. Students also had the opportunity to discuss their feedback face‐to‐face with their assessor.

Written feedback consisted of two elements. Firstly, in‐text comments, which were usually short (up to a few sentences, but often only a few words each), addressed a specific section of the assignment, and were placed close to the relevant text within the assignment (see Appendix S1). The second element was a feedback summary, which usually consisted of at least a paragraph, provided the markers’ overall view on the students’ work and was usually placed either at the end of the assignment or into a separate section (see Appendix S1).

The written feedback (which had been identified by the students as good) was downloaded and examined. The feedback summary was analysed through thematic analysis [35]. The quantity and content of the in‐text feedback comments was analysed according to Voelkel and Mello [36] as follows (e.g. see Appendix S2):

#### Feedback type

In‐text comments were allocated to one of the following three types:

- Content (addressing errors, misconceptions or missing content in the context of the subject of life sciences).
- Writing skills (expression, grammar, spelling, referencing format, structuring, argumentation).
- Motivational (praise for things done well). Note that although motivational comments sometimes could have been allocated to one of the other two types instead, it transpired that motivational comments were often too unspecific to be allocated. Therefore, it was decided to keep ‘motivational’ as a separate type.

#### Feedback depth

Following the methodology of Glover and Brown [37], the depth of each in‐text feedback comment was also established:

- Level 1: Acknowledgement (This kind of comment directs the student to a mistake or weakness but without offering further advice).
- Level 2: Correction (The comment not only highlights a mistake or weakness but also provides advice on how to address the problem).
- Level 3: Explanation (This kind of comment provides reasons why a particular word/sentence would be wrong in this context, or why the student should provide more information).

#### Further feedback characteristics

In addition to the above, we introduced three further characteristics, based on what has been previously suggested as hallmarks of good feedback (e.g. [38]). In contrast to the feedback types and depth described above, comments could be assigned to none, one or more than one of the following categories:

- Easy‐wins (comments that could easily be addressed by following the marker’s suggestion, without the need for further work).
- Specific (it was clear what the feedback referred to, and enough detail to follow up the comment was provided).
- Feedforward (feedback that could clearly be useful for future assignments).

### Interviews

Following the feedback analysis, we invited all students who had sent us examples of good feedback to be interviewed. The interviews aimed to complement the feedback analysis by exploring the students’ views on good feedback characteristics in more depth. Seven students were interviewed by two of the authors of this study, LVM (*N* = 3) and SV (*N* = 4) between February and March 2017, and the interviews were recorded and transcribed. Interviews were semistructured and lasted for about 30 min each. The core interview questions were

1. With regard to feedback, what is important for you?
2. How would you define ‘good feedback’?
3. What makes you engage with feedback?
4. How much does the mark affect your engagement with the feedback?

The transcripts from the interviews were sent to the interviewees who were given one week to correct any mistakes or withdraw part or all of their contributions. All participants were happy with the transcript, and one added additional comments to it. The transcripts were then analysed by one author of the study, TVA.

### Focus group

In addition to the feedback analysis and interviews, we wanted students to cooperatively formulate what ideal feedback looks like. A nominal focus group [39] was considered the best approach to achieve this. An email was sent out to all second‐ and third‐year students inviting them to participate in a focus group. Six students took part in the session which took place in May 2017 and was facilitated by one of the authors (TVA). The 60‐min session was recorded and transcribed. The session was structured as follows:

1. Introductions/consent/ground rules
2. Post‐it task 1: Students write down thoughts about ‘What does ideal feedback look like?’
3. Group discussion
4. Post‐it task 2: Students write sample feedback sentences that they would like to see on written assignments as if they were lecturers
5. Group discussion
6. Final summary task: ‘Write your top 5 wish – list for written feedback in ranked order’

The results of the focus group were summarised by TVA, and the summary was sent to the participants for comments or corrections. All of them were happy with the summary.

## Results

The first part of this study aimed to identify common characteristics of written feedback samples perceived by students as good feedback. Therefore, we asked second‐ and third‐year students if they felt that they received good feedback for a particular written assignment. Although 61 out of 820 students responded initially, not all returned a questionnaire, and not all assessment pieces could be included in the study (e.g. because the feedback was not accessible online in Turnitin). In total, 51 assessment pieces were analysed. Marks for the assessed pieces of work ranged from 58% to 87%, indicating that most respondents were high achievers (see Table 1).

**Table 1:** Respondents.

	Second year (essay)	Third year (literature review)	Total
Initial number of respondents/response rate	23/5.6%	38/9.3%	61/7.4%
Number of students who returned a questionnaire	23	23	46
Number of assessment pieces analysed	21	30	51
Marks average	68%	70%	69%
Marks range	58–87%	58–82%	58–87%

### Feedback analysis

#### Assessors’ in‐text comments

In total, 780 in‐text feedback comments across 51 assessment pieces were analysed (see Table 2). This related to an average of 11 (second year) and 18 (third year) comments per marked assignment, respectively. With regard to feedback types, over half of the comments in both assignments related to writing skills, about a third were about subject content, and only around a tenth of the comments consisted of praise (motivational). Analysing the depth of the comments, we found that in the second‐year essay, the most common feedback depth was at level 1 (acknowledging a mistake or omission), making up nearly half of the comments. In the third‐year literature review, level 2 feedback (correction) was the most common. Further analysis showed that in both assignments, the vast majority of the feedback comments were specific, being detailed and clearly indicating the instance where the feedback related to. Easy‐win comments were less frequent, and only a small proportion provided feedforward that might be applicable for future assignments.

**Table 2:** Analysis of assessors’ in‐text feedback comments^a^.

	Second year (essay) *N* = 21	Third year (literature review) *N* = 30
Number of in‐text comments		
Total	237	543
Average (per student)	11.3	18.1
Median (per student)	11.0	16.5
Min‐max	0–37	0–67
Number of pieces of work with no in‐text comments	1	2
In‐text comments characteristics (%)		
Feedback type		
Content	32	37
Writing skills	55	54
Motivational	13	8
Feedback depth		
Level 1 (acknowledgement)	49	31
Level 2 (correction)	23	40
Level 3 (explanation)	28	29
Additional feedback characteristics		
Specific	77	89
Easy‐wins	34	41
Feedforward	24	9

^a^ For further explanation, please refer to the methods section. Note that percentages for feedback types do not always add up to 100% because a small number of comments could not be allocated to any category. Additional feedback characteristics exceed 100% because comments could be allocated to more than one characteristic.

#### Assessors’ feedback summaries

Just over half of the second‐year essays and 73% of the third‐year literature reviews received a feedback summary from their assessor (see Table 3). The average length of the summaries differed between second and third years (46 versus 70 words, respectively), and this difference becomes even more pronounced when considering the median (12 versus 55 words). In both assignments, where summaries were provided, almost all of them contained praise and suggestions for improvement that could be useful for future assignments. Over half of the summaries contained a justification of the mark, but only a small proportion referred to the marking criteria.

**Table 3:** Analysis of assessors’ feedback summaries.

	Second year (essay) *N* = 21	Third year (literature review) *N* = 30
Number of pieces of work with feedback summary	11	22
Total number of words in summaries	956	2096
Average (words per summary)	45.5	69.9
Median (words per summary)	12	55
Min‐max (words per summary)	0–265	0–336
Containing praise (%)	90.9	95.4
Containing suggestions for improvement (%)	90.9	100
Justification of mark (%)	63.6	54.5
Reference to marking criteria (%)	18.2	4.5

#### Variation between assessors

**Fig. 1:**
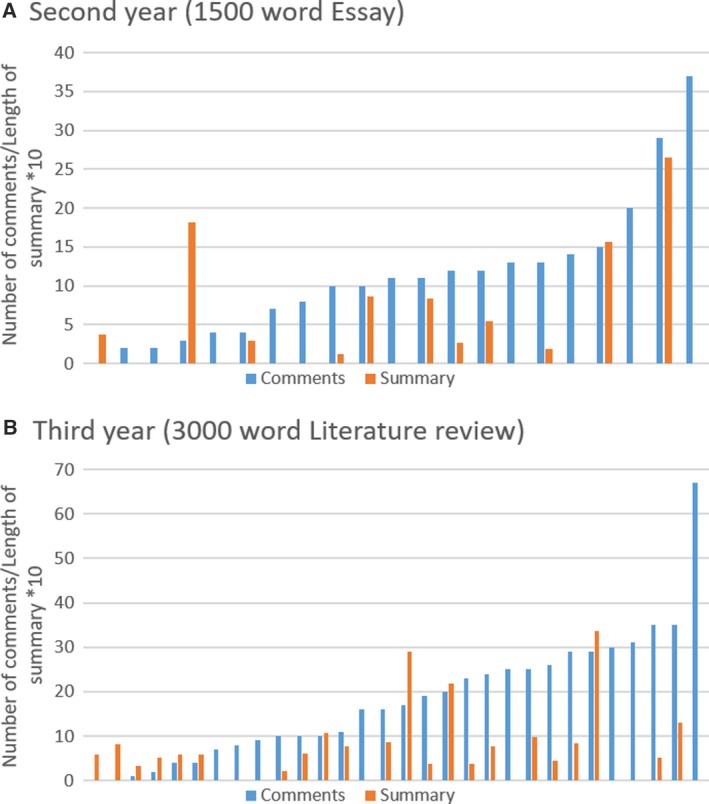
Number of in‐text comments and length of feedback summary for each piece of work. The length of the summary is expressed as the number of words divided by 10. (A) Second‐year essay (*n* = 21), and (B) third‐year literature review (*n* = 30).

The numbers shown in Tables 2 and 3 already indicate the huge variability of the feedback provided for individual pieces of work, both in terms of quantity and quality. The number of in‐text comments on individual pieces of work varied widely, from zero (in three cases) to 67 per marked assignment. Similarly, the length of the feedback summaries varied from zero to 336 words (Fig. 1).

**Fig. 2:**
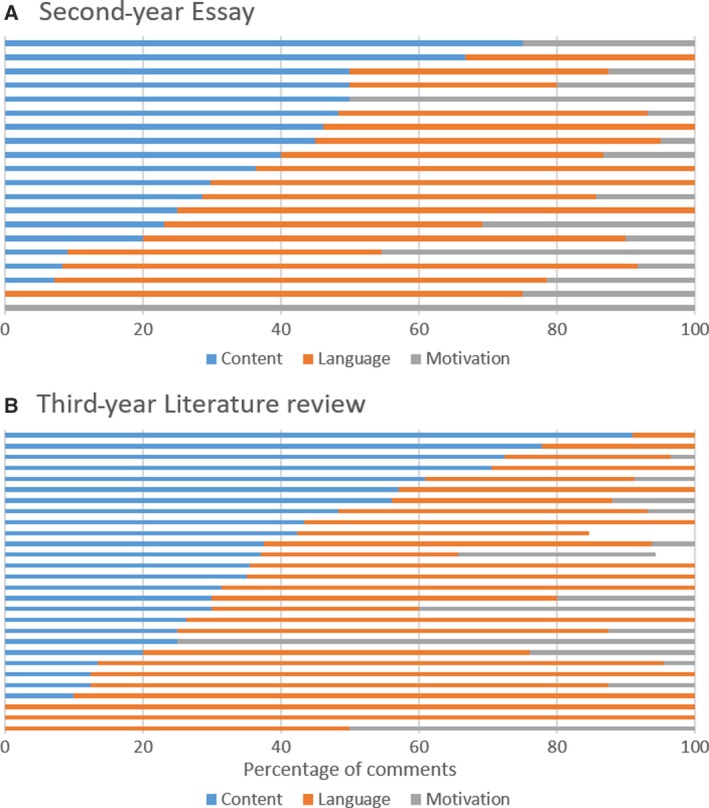
Percentage of in‐text comments in each category for each individual piece of work. Feedback that did not contain any in‐text comments has not been included (Note: a few comments could not be allocated a category and/or level, therefore not all bars reach 100%). (A) Second‐year essay (*n* = 21), and (B) third‐year literature review (*n* = 30).

**Fig. 3:**
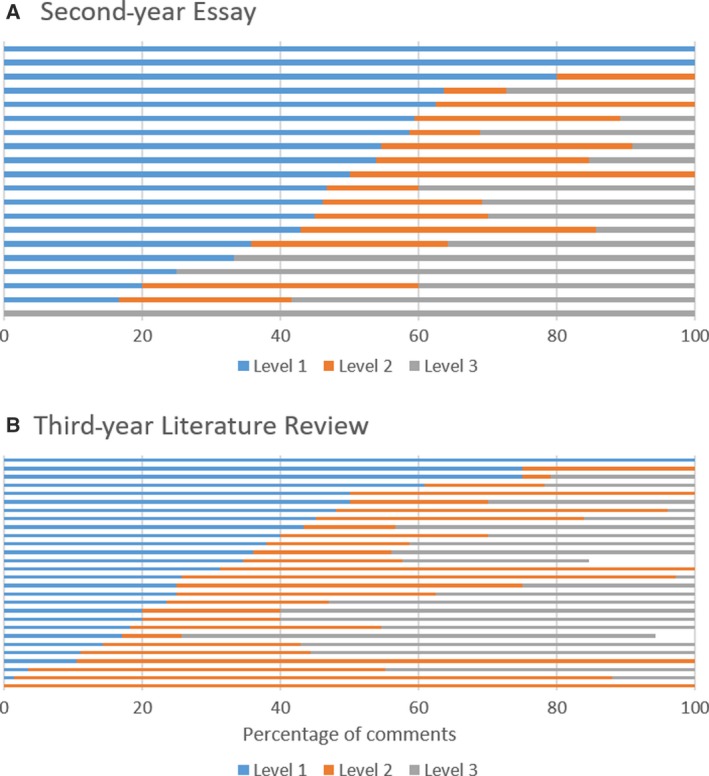
Percentage of in‐text comments at each level for each individual piece of work (Level 1 = acknowledgement, Level 2 = Correction, Level 3 = Explanation). Feedback that did not contain any in‐text comments has not been included. (Note: a few comments could not be allocated a category and/or level, therefore not all bars reach 100%). (A) Second‐year essay (*n* = 21), and (B) third‐year literature review (*n* = 30).

There was also a large variation in the composition of in‐text feedback (i.e. the ratio between content, writing skills and motivational comments as well as between the different levels of depth). Comments related to writing skills made up the majority in 65% of the assignments, whereas ‘subject content’ was the most frequent type of comment in 30%. Only fewer than 5% of the cases focused primarily on motivational comments. 44% of the assignments did not contain any motivational comments at all (Fig. 2). As for the depth of in‐text feedback, level 1 (acknowledgement) was most common in 44% of the assignments, level 2 (correction) in only 16% and level 3 (explanation) in 28%. 26% pieces of work did not contain any explanatory comments at all (Fig. 3).

**Fig. 4:**
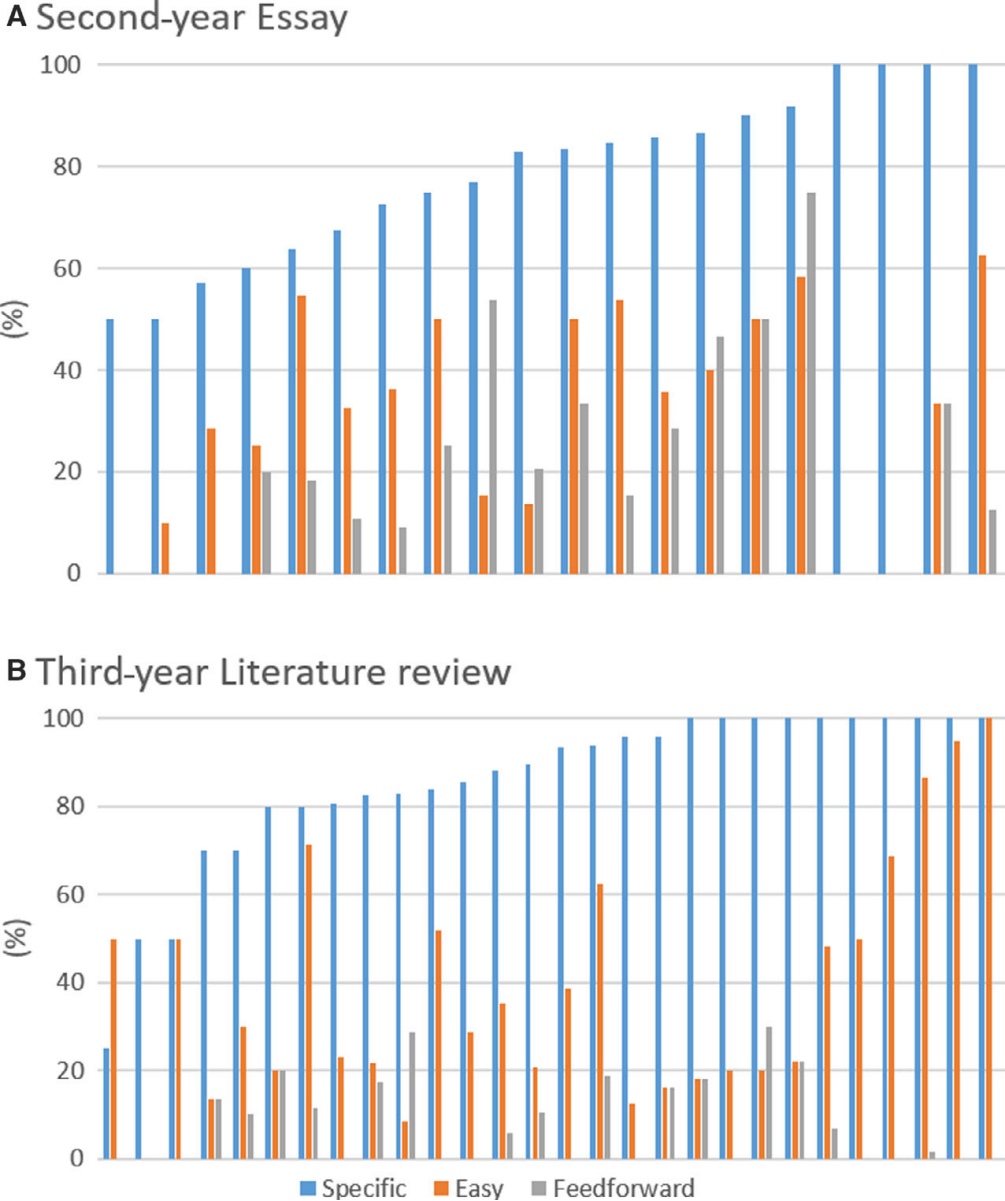
Percentage of in‐text comments that were specific, easy or feedforward in each individual piece of work (Note that feedback comments could be characterised as neither, one or more of the three categories ‘specific’, ‘easy‐win’, ‘feedforward’). (A) Second‐year essay (*n* = 21), and (B) third‐year literature review (*n* = 30).

Comparing the frequency of the additional in‐text feedback characteristics (specific, easy‐win, feedforward), there was much less variation. In almost all assignments (96%), the majority of the in‐text comments were specific. In fact, in 70% of the assignments, 80% or more of all in‐text comments were classified as specific. There was no assignment that did not contain any specific feedback. However, easy‐win feedback was not as frequently found: in only 4% of the cases was easy‐win feedback the most common category. Still, in 35% of the assignments at least half of the in‐text comments were classified as easy‐wins. By contrast, feedforward comments were consistently infrequent with only 4% of the assignments containing 50% or more of their in‐text feedback comments assigned to this category. 38% of the feedback pieces had no feedforward comments at all (Fig. 4).

### Students’ views

The second part of the study focused on students’ views of what constitutes good feedback. We used a questionnaire, interviews and a focus group.

### Questionnaire

Altogether, 46 questionnaires were analysed. When asked to explain in their own words what they found good about the feedback they had received, 84% of the respondents said that their feedback had been specific, 45% liked that the feedback comments were easy to follow‐up, and 31% thought that their feedback would help them in future assignments. In addition, 16% mentioned that in this assignment they were able to discuss their feedback face‐to‐face with their marker. When asked how their feedback could be even better, the most common suggestion was that the feedback could be more specific, but only 16% said this. 10% thought that the feedback could refer more to the marking criteria, and 8% would have liked examples of good work. Regarding the question what they did in response to their feedback, all respondents said that they had addressed those comments that were easy to implement to improve their essay or future final report.

### Interviews

Most of the seven interviewed students appeared to be high achievers. All of them had a keen interest in receiving feedback, and they appreciated that feedback will be constructive in order to help them improve. Even if they were reticent to engage with the feedback directly after receiving it for a day or so, they would not be discouraged by a high number of comments and suggestions for improvements.

Interviewees consistently said that good feedback should specifically and clearly point out what they have done well, but also to tell them what they have done wrong. For the interviewed students, it was also important that feedback tells them how they can improve or ‘fix it’. A number of students commented on verbal, face‐to‐face discussions being helpful after receiving their written feedback. This would allow for clarification and discussion of details. Students said they would generally engage least with their written feedback when they received their expected grade. Where they underachieved, they had a look at their feedback to see what they have done wrong, or if they got an unexpected high grade, then they looked at their feedback to find out why (Table 4).

**Table 4:** The main themes identified in student responses to the main interview questions. Seven students were interviewed, and the names listed here are fictitious.

*What is good feedback, and what is important for you?*	
Apple	What you have done well and what to change; suggestions for improvements; Knowing what you have done well – gives confidence boost. Some specific advice on structuring. Personal/face‐to‐face discussion after written feedback is helpful and preferable to written feedback [alone]
Claire	Good feedback ‘tells you what you did right, but also says what you got wrong and how to improve’. The student struggles with writing and so appreciates help and feedback on language and structure.
Daisy	Tells you how to improve. Rubric not enough (‘doesn’t tell you how to move from good to excellent’)
Lisa	Reinforce the positives, reinforce what you need to improve on. Pick up details – so for feedback to be specific; context, detail, exemplify. Suggesting ways to move from bad to good. Integrating rubric with in‐line comments. Student likes tutor to be available for further discussion
Martin	Identify everything what I have done wrong and tell me how to fix it. Student finds verbal discussion of feedback very helpful.
Naomi	For feedback to be clear, to know exactly what’s gone wrong and what you can improve. When comment explains something that help student realise [themselves] what went wrong. Or when a comment helps student to rethink a point made. Just to get one word, for example ‘awkward’ does not constitute good feedback, not specific enough
Simone	Having the ability to apply it; specific feedback might not always help, ‘I need to hear why to improve that way’. The feedback process is an exchange whose purpose is to deepen students’ understanding. Praise very helpful as can point out what’s good, for example why a structure was good. Feedback is about learning to be a scientist – that helps students understand what scientific writing is (why it’s done in certain ways). Instance of less helpful feedback – given on a draft assignment but unclear how could have improved so final version was only few marks better whereas aimed for ‘an exemplary piece of work’. Seeing bad and good examples, both are useful
*What makes you engage with feedback, and how does your mark affect your engagement?*	
Apple	Engages more with feedback on drafts; helpful: feedback sessions discussing model answers. If mark is what expected engagement is less; if lower or higher then would look at feedback more. Strong on valuing formative versus summative feedback
Claire	Prefers written feedback and typed (legibility) – too shy for seeking face‐to‐face feedback
Daisy	Engages with feedback when discussed in a group. Discomfort when discussing individual feedback in front of a group
Lisa	Points out interface/usability issues with student view of Turnitin
Martin	Looks at feedback when gets lower than 70% mark. Suggests that students are given 2% for engaging with feedback, for example summarising how they would improve based on feedback, etc.
Naomi	More likely to engage with feedback if got lower than expected mark. But also wants to know what they have done well (in specific terms). Discussion with tutor was helpful to highlight points. Prefers short and quick feedback, for example in a module last year only got feedback at the end when they couldn’t improve – would have been more useful to get weekly feedback so could have then improved week on week
Simone	Engages most when she can apply it (when it tells what/why to improve) and when it’s formative (can improve for final) Very high engagement – always on the lookout for how to improve, seeks out most feedback opportunities, talks to tutor, etc. Detailed feedback more important than timeliness If not told the ‘why’ to improve – this results in disengagement (even if feedback is specific and detailed)
*Feedforward (feedback linking between modules)*	
Apple	Wouldn’t probably link feedback and use it for other modules, sees it as more separate
Claire	Yes, some feedback, for example, on diagrams can be used in other assignments
Lisa	Feedback can be isolated. Depends on assignment type. More in essays but less so for, for example, online tests
Simone	Advice on written communication skills can be integrated into other modules
*Taking negative feedback*	
Apple	Depends on context – would close/reopen – but doesn’t seem much bothered about it emotionally
Daisy	Optimistic person so knows to handle feedback constructively. Really values feedback as knows it’s for her to improve
Lisa	Low mark can hurt pride – reinforcement of positives can give you motivation
Martin	Understands that critique is constructive, doesn’t get emotionally negative on seeing lots or negative feedback
Naomi	If you have a chance to rework draft after feedback, then comments are very useful. Not bothered by lots of red pen as used to it from doing languages at school All negative can be bad – as you might think all what you have done is wrong – so need to have some positive reinforcement too
*What advice on feedback would you give to new staff*	
Apple	Make feedback personalised. What specifically it was that I did well and what I can do to improve
Simone	Highlight good examples, but also bad ones. Tell us why we should improve it Engage with students in dialogue as to what their target mark is – so that appropriate support and encouragement could be given

### Focus group

Six students took part in the session, and none of them had contributed to the other parts of this study. The focus group explored students’ general views on feedback (for typical quotes, see Table 5). The first discussion topic (What does ideal feedback look like?) revealed an additional aspect to the feedback discussed in the feedback analysis and interviews, namely the importance of feedback during the drafting stage of an assignment. Once students received the assignment brief, they would start working on the assignment using the marking criteria to see how to shape the assignment. It is at this drafting stage, when students would value feedback most. This feedback helps them to get on the right track, making sure that they are compiling an assignment that was envisaged by those who set it.

**Table 5:** Focus group – Student views on what makes feedback ‘good’.

Theme	Typical quotes
*Feedback at the drafting stage*	
Discussions with tutor/ question setter	‘I think that something that might be obvious to a lecturer isn’t necessarily obvious as a student’ ‘As a student you kind of sometimes feel left in the dark, like you’ve got no idea what they want’ ‘Can you just give me a little list of things that you like or that really bug you?’ ‘it really helped us to be able to sit and talk with her rather than just following instructions given to us at the start because she could then tell how we were getting confused and could rephrase stuff’
Model answers	‘We had model essays/ reports and they’ve been really insightful in knowing what you need to progress to a higher grade’ ‘… so we actually know what the kind of detail that they would like is, because otherwise I could find myself being too brief or too specific and the report doesn’t call for that’
Making sense of the marking criteria.	‘when you’ve only got your own essay to look at and you’re criticising yourself, it’s really, really hard to place it on the rubric because you’ve got nothing else to compare it to’ ‘[the rubric] has things like ‘it needs specific detail’ or ‘very specific detail’ or ‘not enough detail’, and when you read that it’s very ambiguous and you don’t know what it means’
*Feedback after grading has taken place*	
Justifying the mark	‘If I’ve got a lower grade, why have I got a low grade and what did I need to do to get the higher grades?’ ‘What makes my essay a 61 or a 62 and not a 60 or a 65? How could I boost it up a bit more to get it into the next grade?’
Positive reinforcement	‘Very good paragraph of being able to tie different topics together.’
How to improve	‘In order to get a higher grade, you should be doing …’
Feedback should be detailed and specific	‘you can’t tell if it’s about the last sentence or the entire paragraph, so it’s nice when they say good and then what they mean and don’t just put a random “good”’
Honest and constructive criticism	‘don’t be afraid to criticise because sometimes you might be surprised that maybe the student just knows that, that that’s what is actually wrong with it’
Content and structure are more important than spelling and grammar	‘Basically all they’d done is just corrected all my grammar in it which I understand is really important, but at the same time I don’t feel I found out what was good to actually improve and take on to further things’

When asked about their idea of ‘ideal feedback’ after grading, students agreed on the following key characteristics (wish list), presented in order of students’ preference:

- Justification of the mark: students find it useful to know how the marker arrived at their grade. This is about being aware of what the grading means and how to achieve better grades.
- Positive reinforcement: emotionally, students value positive reinforcement of work done well. It makes it easier for them to receive criticism. Another reason for students signalling a need for praise is that they felt it validated their work. Such reinforcement has the power of making explicit what it is that they are doing is positive; they are not always aware if they are doing something well. It helps them ‘learn’ what it is they are doing well and then being able to reuse that strategy again in other places.
- How to improve: Ideal feedback for students includes instructions how they can improve their work in specific ways. Students also find it valuable if we tell them the reasons why the improvement is necessary. This is particularly helpful if it is linked to the marking criteria.
- Feedback should be detailed, clear and specific. Students need to be told exactly what they have done well and why; or details of what is lacking and why. Students said that if the feedback was just saying ‘good’, they would not be able to tell what it is that they did well. Another important aspect is that comments specify the location in the text that the feedback refers to.
- Honest and constructive criticism: students emphasise that feedback only works if it is honest. Sometimes students themselves know if their work is not as good, and in this case, reinforcing this is justified.
- Advice on improving content and structure or argumentation of assignments is seen as much more valuable than having grammatical or stylistic errors pointed out to them.

## Discussion

The aim of this study was to find out what our students want from feedback, and based on that, to develop a guide for markers. To achieve this, we firstly asked students to share with us an example of good written (electronic) feedback they had received. We did not specify what we meant by ‘good’, other than that they should have found the feedback useful. We then analysed the feedback examples to see if there were any common characteristics that would help define good feedback. We found that the quantity of the written feedback varied hugely. For example, students with as little as 40 or 50 words of feedback summary (and no in‐text comments) still thought their feedback was good. Clearly, it was not a matter of ‘the more the better’ for the students. This echoes Lilly *et al*.’s [24] finding that the length of feedback does not in itself matter so much to students.

With regard to the composition of the good feedback provided as in‐text comments, the majority of these comments related to writing skills, followed by subject content‐focused comments, and only a relatively small percentage was praise. The prevalence of writing skills‐related feedback confirms Hyland’s study [40], which also found that most feedback given to students was about ‘form’ (including structuring, grammar). But the small proportion of praise found in our study was surprising, as several previous studies indicate students’ desire for praise (e.g. [28, 41]). When Orsmond and Merry [42] analysed feedback comments, they indeed found that praise was the most frequent form of feedback. On the other hand, according to Dawson *et al*. [38] only a minority of students think that the purpose of feedback is to motivate students. In our study, it is possible that students’ desire for praise was satisfied via the summary comments (rather than the in‐text comments), almost all of which contained some form of praise.

We also analysed the ‘depth’ of in‐text feedback comments. Although there was a lot of variation between markers, many comments were either an acknowledgement of a mistake, or a correction; only a small number contained explanations. Glover and Brown [37] similarly analysed the depth of written feedback to students in biological and physical sciences. They found that the majority of comments addressed omissions and language issues, whereas only about 11% of comments consisted of tutor clarification. Although the latter study did not focus on feedback that was identified as good by students, this outcome indicates a tendency towards low ‘depth’ feedback. Interestingly, the study found no or little relation between the depth of feedback and the grade [37]. In our study, the majority of students did not seem to need or expect any explanations. Instead, they found it important to know how to improve. Other studies also found that students wanted constructive criticism that contained suggestions for improvement, for example by correcting errors [29, 42]. As pointing out errors and suggesting a correction would enable students to amend their drafts, it may not be surprising that students liked this form of feedback.

The frequency of the additional feedback characteristics (specific, easy‐wins, feedforward) was less variable than the depth. Firstly, almost all in‐text comments were specific. This ties in with other studies emphasising the importance of detailed and specific feedback comments (e.g. [16, 38]). This finding also mirrors the results of the questionnaire, where most students liked the fact that the feedback was specific. Surprisingly, much less feedback was classified as easy‐wins. Our expectation had been that students would favour easy comments, that is comments that made it easy for them to correct mistakes, for example by providing the correct information without the need for the student to do any further work on it. However, students might have a different view on what are easy‐wins. In the questionnaire, many students said that they liked the fact that comments were easy to implement and that they used them to improve their work. It is possible that students found comments easy to implement because they were specific, but were not relying on markers to provide the ‘easy fix’.

Another surprising result was that relatively few comments were classified as feedforward, that is potentially useful for future assignments in other modules. In the second‐year assignment, only 24% of the in‐text comments were feedforward. This roughly tallies with the questionnaire where only just under a third of the second‐year students said that the feedback would help with future assignments. In the third‐year assignment, even fewer (9%) comments were feedforward. We would have thought that the ability to use feedback to improve future assignments would be more important to students. Many studies identify feedforward as an essential part of effective feedback (e.g. [43]). However, it is possible that because both assignments in this study were directly related to another submission in the same module, students were focused on specific improvements, and not so much on other, unrelated assignments. Indeed, 65% of the second‐year students resubmitted their essay and all of them achieved a higher mark for their second submission (average mark increased from 65% to 73%), indicating that the students had enough information to be able to improve their essay. This ties in with Reimann *et al*. [44] who found that feedforward practices are often situated within current modules. In our study, it is noteworthy that even in the focus group which addressed feedback more generally, feedforward was not mentioned as an important ‘ideal feedback’ characteristic.

Overall, one of the most surprising outcomes of our analysis of good feedback examples was how much the good feedback varied in terms of quantity and many feedback characteristics. However, Poulos and Mahoney [27] state that students do not have a homogenous view on what effective feedback actually is. Orsmond and Merry [28] found that low‐ and high‐achieving students differ in their perception of feedback, which may be related to a higher degree of self‐regulation in high achievers [19]. However, a recent systematic review on feedback found conflicting results and concluded that engagement with feedback may be affected by prior experience and not only depend on students’ academic skills [45]. Bjork *et al*. [46] discuss that students’ judgement of their own learning is highly subjective and influenced by intuitions and beliefs. What is more, Pitt and Norton [47] argue that students vary in their emotional maturity and this, together with their grade expectations, might affect their views on and engagement with feedback. According to Lizzio and Wilson [48], feedback is most likely found to be effective by students if it is developmental, encouraging and fair. All comments that were analysed in our study could be characterised as such. Neither in‐text nor summary feedback in the examples submitted contained any negative, demoralising phrases, which could have affected students’ willingness to engage with their feedback [47].

The analysis of good feedback examples and the questionnaires was complemented by interviews and a focus group. A consistent theme in all parts of the study was that according to students, good feedback should be specific and detailed. Similarly, information about how to improve was highlighted as important during both interviews and focus group. This is mirrored by the feedback examples that mostly contained information about what students had done wrong and how to correct this. The focus group put ‘justification of mark’ at high priority. Although this was not a strong theme in the interviews, this might be due to the fact that the interviewees were mostly high achievers, whereas a justification of the mark may be more important for students who achieved disappointing results [49]. Looking at the analysed in‐text comments, these rarely justify the mark, but more than half of the feedback summaries provide this. Positive reinforcement (praise) again was identified as important in the focus group, but less so in the interviews, and very few in‐text comments praised. But as almost all the general comments contained praise, this might again have fulfilled the students’ needs.

The present study has a number of limitations that need to be kept in mind when trying to generalise the findings. Firstly, this study mainly reflects the views of students who were engaged and who had achieved good marks, ranging from 58% to 87%. We did not hear from weak or failing students, and we did not see any feedback provided to them. This may not be surprising, as Jones *et al*. [50] point out that high achieving students are more likely to complete questionnaires about feedback. However, this means that we cannot draw any conclusion regarding feedback that weaker students might find useful, and therefore, the findings of this study might not be representative of all students. Secondly, this study only involved second‐ and third (final)‐year students, and there is a possibility that students in other years have different views. Thirdly, this study only involved students from the life sciences, and we do not know if the results also apply to students in other disciplines. Fourthly, we did not collect information about student characteristics (e.g. English as second language or specific learning disabilities), and therefore, we cannot comment on feedback characteristics that specific student groups might have found useful.

In addition, it should be remembered that both assignments had an inbuilt option for improvement (resubmit, use for final), which is not necessarily typical for written assignments. Some students’ written feedback was complemented by face‐to‐face discussion, so they might have confounded this interaction with their written feedback, rating their feedback overall as good although the written feedback alone might not have been sufficient. Also, there are other ways to analyse feedback: for example, Kumar and Stracke [51] used feedback categories based on the three functions of speech (referential, directive or expressive). And finally, this study is entirely based on students’ perception of what constitutes good feedback. We did not analyse any impact of feedback on students’ learning, and therefore, we cannot comment on the actual effectiveness of the feedback.

## Conclusions and recommendations

This study has used a combination of mixed methods (drawing from four data resources: feedback analysis, questionnaire, interviews and a focus group) to identify characteristics of good feedback based on student perceptions. This high degree of triangulation allows us to suggest a list of recommendations for staff wishing to compose written feedback on assignments that students find useful. It is clear from our study that for feedback to be perceived as good by students, quality is more important than quantity. What is also clear is that there are many ways of providing good feedback. Although this study does not provide a ‘model method’ for feedback, it does contribute to the development of feedback models by presenting aspect of feedback that are valued by students.

According to students, good feedback:

1. Is detailed, clear and specific and relates to specific areas of their work.
2. Tells students exactly how to improve. Some students also find it valuable if we tell them the reasons why the improvement is necessary.
3. Is honest and constructive.
4. Includes positive reinforcement. Praise for aspects of the work done well makes it easier for them to receive criticism. It also reinforces positive aspects of their work, explaining what it is they’ve done well and should keep doing.
5. Justifies the mark. Knowing how the marker arrived at their grade helps students understand what the grading means and how to achieve better grades.

Although advice on subject content, structure or argumentation is seen as more valuable than having grammatical or stylistic errors pointed out to them, some students also struggle with language and find corrections helpful.

In addition, students highly appreciate a feedback opportunity during the drafting stage. This enables them to clarify the task and make sure they are on the right track.

## Conflict of interest

The authors declare no conflict of interest.

## Author contributions

SV conceived, and SV and LVM designed the project. SV, LVM and TVA collected the data; SV and TVA analysed the data. SV wrote the manuscript, and LVM and TVA commented on multiple versions of the manuscript.

## Supporting information


**Appendix S1.** Screenshot of an example assignment in Turnitin feedback studio. An example of an in‐text comment is shown. Assessors can highlight specific text passages and place a comment directly within the text of the assignment. Summary comments are usually provided in a specific section or placed at the end of the assignment.
**Appendix S2.** Examples of identified feedback types, depth and characteristics used for the analysis of in‐text feedback comments. (Depth: 1 = acknowledgement, 2 = correction, 3 = explanation, see Methods for detail). For each comment, it is indicated if it was also classified as ‘specific’, ‘easy’, and/or ‘feedforward’.
