# Supplementary material for: Students tell us what good written feedback looks like
Source: FEBS Open Bio. 2020 Mar 30;10(5):692–706. doi: 10.1002/2211-5463.12841 (PMC7193163; doi:10.1002/2211-5463.12841)
Supplement: Supplementary file 1 — Appendix S1. Screenshot of an example assignment in Turnitin feedback studio. An example of an in‐text comment is shown. Assessors can highlight specific text passages and place a comment directly within the text of the assignment. Summary comments are usually provided in a specific section or placed at the end of the assignment. Appendix S2. Examples of identified feedback types, depth and characteristics used for the analysis of in‐text feedback comments. (Depth: 1 = acknowledgement, 2 = correction, 3 = explanation, see Methods for detail). For each comment, it is indicated if it was also classified as ‘specific’, ‘easy’, and/or ‘feedforward’. [file FEB4-10-692-s001.docx]

**Supporting information**


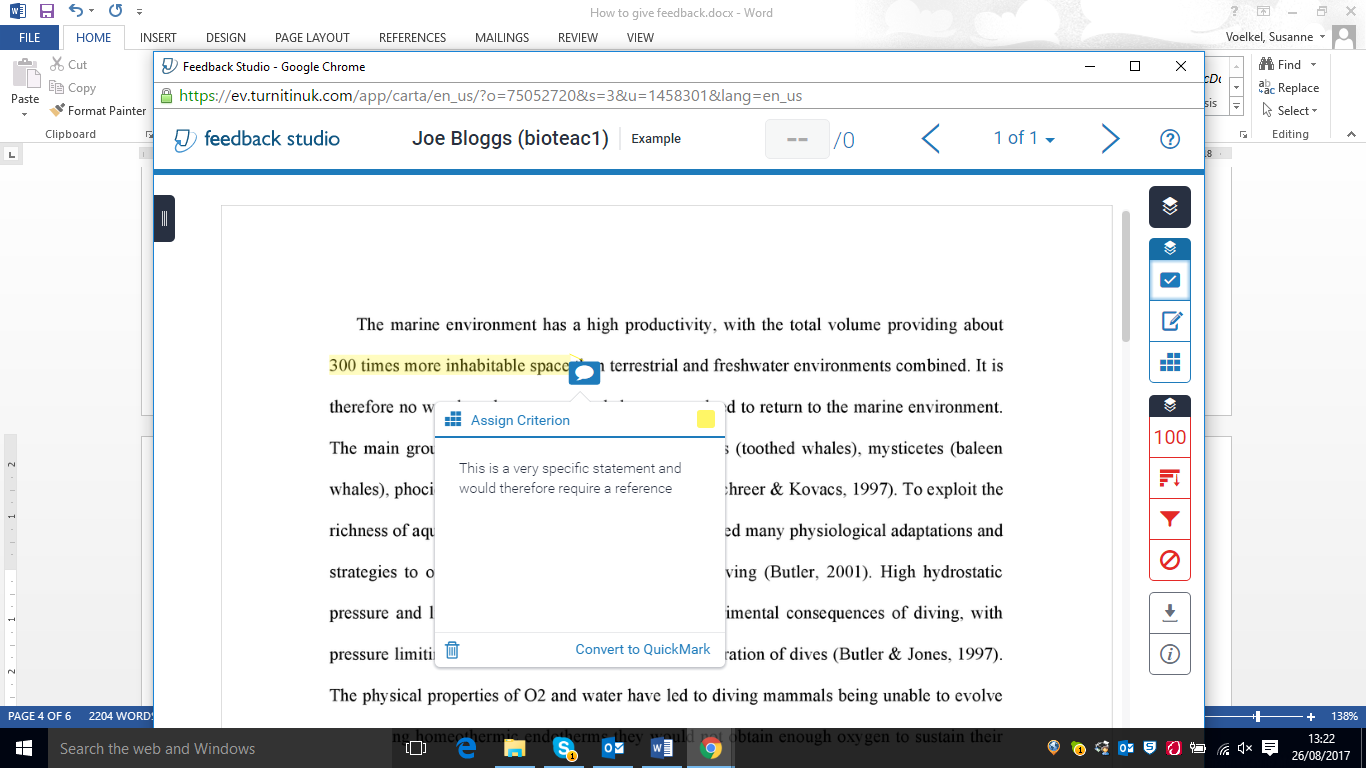


S1: Screenshot of an example assignment in Turnitin feedback studio. An example of an in-text comment is shown. Assessors can highlight specific text passages and place a comment directly within the text of the assignment. Summary comments are usually provided in a specific section or placed at the end of the assignment.

| S2: Examples of identified feedback types, depth and characteristics used for the analysis of in-text feedback comments. (Depth: 1= acknowledgement, 2= correction, 3=explanation, see Methods for detail). For each comment, it is indicated if it was also classified as ‘specific’, ‘easy’, and/or ‘feedforward’. | | |
| --- | --- | --- |
| Feedback types | Depth | Example comments |
| Content | 1 | ‘This needs a reference’ (specific)  ‘illogical given what follows (also wrong)’  ‘Is this correct as the structure of all tissues?’ (specific) |
|  | 2 | ‘Receptor (TNFR1), not TNF1.’ (specific, easy)  ‘More correctly, in populations with different forces of infection’ (specific, easy) |
|  | 3 | ‘BH3 only proteins are repressed by the anti-apoptotic members and not the other way around. The BH3 only members are released from their anti-apoptotic counterparts, which is followed by activation and MOMP.’ (specific)  ‘Really interesting, but I want to know which crops at this point.’ (specific)  ‘You need to explain more about how the data were collected’ (specific, feedforward) |
| Writing skills | 1 | ‘Why is it capitalized?’ (specific, easy)  ‘Bad citation format’ (specific, easy)  ‘Minor grammar glitch here’ |
|  | 2 | ‘You need to maintain past tense throughout’ (specific, easy, feedforward)  ‘This should be a comma, not a semicolon.’ (specific, easy)  ‘You could just say 'adapted from...(ref )' (specific, easy) |
|  | 3 | ‘Incomplete citation, you always need to give the year of publication so that the reader can find it’ (specific, easy, feedforward)  ‘One thing I would suggest to improve further is to make better use of your figure. I.e. Put the figure adjacent to the text that  describes the same thing that the figure illustrates and refer to your figure in text.’ (specific, easy, feedforward) |
| Motivational | 1 | ‘good introduction’ |
|  | 2 | ‘good link to the next paragraph’ (specific, feedforward) |
|  | 3 | ‘The first couple of paragraphs are a nice introduction to the report and the importance of research into CAM, well done.’ (specific, feedforward)  ‘This is really good- you're commenting on what needs doing as well as what has already been done. This shows good analysis/ synthesis/critique skills.’ (specific, feedforward) |
